# Favipiravir Does Not Inhibit Chikungunya Virus Replication in Mosquito Cells and *Aedes aegypti* Mosquitoes

**DOI:** 10.3390/microorganisms9050944

**Published:** 2021-04-27

**Authors:** Sofie Jacobs, Lanjiao Wang, Ana Lucia Rosales Rosas, Ria Van Berwaer, Evelien Vanderlinden, Anna-Bella Failloux, Lieve Naesens, Leen Delang

**Affiliations:** 1 KU Leuven, Department of Microbiology, Immunology and Transplantation, Rega Institute for Medical Research, Laboratory of Virology and Chemotherapy, Herestraat 49, B-3000 Leuven, Belgium; sofie-jacobs@kuleuven.be (S.J.); wang.lanjiao@kuleuven.be (L.W.); analucia.rosalesrosas@kuleuven.be (A.L.R.R.); ria.vanberwaer@kuleuven.be (R.V.B.); evelien.vanderlinden@kuleuven.be (E.V.); lieve.naesens@kuleuven.be (L.N.); 2 Laboratory of Arboviruses and Insect Vectors, Institut Pasteur, 75015 Paris, France; anna-bella.failloux@pasteur.fr

**Keywords:** favipiravir, T-705, activation, antiviral activity, mosquitoes, chikungunya virus

## Abstract

Favipiravir (T-705) is a broad-spectrum antiviral drug that inhibits RNA viruses after intracellular conversion into its active form, T-705 ribofuranosyl 5′-triphosphate. We previously showed that T-705 is able to significantly inhibit the replication of chikungunya virus (CHIKV), an arbovirus transmitted by *Aedes* mosquitoes, in mammalian cells and in mouse models. In contrast, the effect of T-705 on CHIKV infection and replication in the mosquito vector is unknown. Since the antiviral activity of T-705 has been shown to be cell line-dependent, we studied here its antiviral efficacy in *Aedes*-derived mosquito cells and in *Aedes aegypti* mosquitoes. Interestingly, T-705 was devoid of anti-CHIKV activity in mosquito cells, despite being effective against CHIKV in Vero cells. By investigating the metabolic activation profile, we showed that, unlike Vero cells, mosquito cells were not able to convert T-705 into its active form. To explore whether alternative metabolization pathways might exist in vivo, *Aedes aegypti* mosquitoes were infected with CHIKV and administered T-705 via an artificial blood meal. Virus titrations of whole mosquitoes showed that T-705 was not able to reduce CHIKV infection in mosquitoes. Combined, these in vitro and in vivo data indicate that T-705 lacks antiviral activity in mosquitoes due to inadequate metabolic activation in this animal species.

## 1. Introduction

Favipiravir (T-705; 6-fluoro-3-hydroxy-2-pyrazinecarboxamide) is an antiviral drug that has been approved in Japan for the treatment of pandemic influenza virus infections. It is a nucleobase analog which is converted intracellularly into its active, phosphoribosylated form, T-705-RTP. This active molecule behaves as a pseudo-purine and is incorporated into the growing viral RNA chain by the viral RNA-dependent RNA polymerase (RdRp), which may lead to either chain termination or lethal virus mutagenesis or a combination of both [1,2]. Studies in favor of both hypotheses have been published [3,4,5,6,7].

T-705 not only inhibits influenza viruses, but also a wide range of other RNA viruses, including alphaviruses (reviewed in [8]). The conserved F1 motif in the viral RdRp has been suggested to play a key role in the broad-spectrum antiviral activity of T-705 against positive-sense single-stranded RNA viruses [9]. T-705 has previously been used off-label for compassionate use in Lassa fever virus infections and has been studied in clinical trials for the treatment of Ebola virus infected patients [10,11,12]. More recently, T-705 has also been studied for the treatment of SARS-CoV-2 infections [13,14].

A re-emerging virus that could be inhibited by T-705 is the chikungunya virus (CHIKV) [3,15]. CHIKV is a mosquito-borne virus that has become a serious public health concern. During the past two decades, it has caused large outbreaks characterized by high morbidity in the form of severe joint pain that can persist for months up to years after the initial infection. Since no CHIKV-specific antivirals are currently available, the repurposing of previously approved broad-spectrum antiviral agents such as T-705 may represent an alternative approach to treat CHIKV infections. We previously showed that T-705 is a potent inhibitor of CHIKV in mammalian cell cultures [3]. The oral T-705 treatment of CHIKV-infected AG129 mice resulted in a decrease in mortality by more than 50% [3]. Moreover, T-705 also inhibited CHIKV replication in the joints in a non-lethal C57BL/6J mouse model [16].

Alternative approaches to control CHIKV (and other arbovirus) outbreaks focus on the mosquito vector. The most common vector-control strategies (reviewed in [17]) involve the use of insecticides, management of mosquito breeding sources and generating mosquitoes that are refractory to arbovirus infection either by genetic modification or infection of the mosquito population with a symbiotic bacterium such as *Wolbachia*. A novel approach that has recently been proposed involves the use of small molecule antivirals to block arbovirus transmission by mosquitoes [18,19]. As CHIKV-specific antivirals are not available yet, we evaluated here whether T-705 has the potential to block the CHIKV infection in mosquitoes.

T-705 requires several steps of metabolic activation, starting with the hypoxanthine guanine phosphoribosyl transferase (HGPRT)-mediated formation of ribofuranosyl 5′-monophosphate (T-705-RMP), followed by two additional phosphorylations to yield the active form, T-705-RTP [1,20]. Previous studies have demonstrated that the antiviral efficacy of T-705 varies depending on the cell line used for in vitro evaluation. This was observed for commonly used mammalian cell lines: Vero, HEK293T, MDCK, A549, and HUH-7 [14,21]. The variable antiviral efficacy of T-705 appears to be explained by the cell line-dependent efficiency to metabolize T-705 into its RTP form, as demonstrated by metabolic studies. Interestingly, mosquitoes were previously found to lack an HGPRT-encoding gene [22], which led us to hypothesize that T-705 cannot be metabolized and thus cannot be activated by mosquitoes. To explore this hypothesis, we assessed the antiviral efficacy of T-705 against CHIKV in *Aedes* mosquito-derived cell lines (i.e., Aag2-AF5, derived from *Ae. aegypti* and C6/36, derived from *Ae. albopictus*) as these mosquito species are the vectors for CHIKV transmission to humans. In addition, we studied the metabolic activation profile of T-705 in these cell lines. To corroborate our in vitro findings in an in vivo model, we determined the impact of T-705 on the infection rate and viral load in CHIKV-infected *Ae. aegypti* mosquitoes.

## 2. Materials and Methods

### 2.1. Cells

Vero cells were maintained in a minimal essential medium (MEM 1X) supplemented with 10% fetal bovine serum (FBS), 2 mM l-glutamine, 0.075% sodium bicarbonate, 1% non-essential amino acids (NEAA), 100 units/mL penicillin, and 100 µg/mL streptomycin at 37 °C with 5% CO_2_. *Ae. albopictus* derived cells (C6/36, obtained from ATCC, CRL-1660) were maintained in Leibovitz’s l-15 medium supplemented with 10% FBS, 1% NEAA, 10 mM HEPES buffer, 100 units/mL penicillin, and 100 µg/mL streptomycin. *Ae. aegypti* derived cells (Aag2-AF5; a kind gift from Prof. Maringer, University of Surrey, Guildford, UK [23]) were maintained in Schneider’s Drosophila medium, supplemented with 10% FBS, 2 mM l-glutamine, and 1% penicillin/streptomycin. Both mosquito-derived cell lines were incubated at 28 °C without CO_2_. For cell culture assays that involved the virus or virus-infected material, the concentration of FBS in the medium was reduced to 2%. All the cell culture media and supplements were obtained from Gibco, Thermo Fisher Scientific (Aalst, Belgium).

### 2.2. Compounds and Viruses

Favipiravir (T-705) was purchased as a custom synthesis product from BOC Sciences (New York, NY, USA) and dissolved in DMSO. Hydroxychloroquine sulfate (HCQ) was purchased from Acros Organics (Thermo Fisher Scientific, Waltham, MA, USA) and dissolved in sterile water.

The CHIKV Indian Ocean strain 899 (GenBank FJ959103.1) was generously provided by Prof. Drosten (University of Bonn, Bonn, Germany) [24]. A virus stock was generated by passaging the isolate on Vero cells.

### 2.3. Antiviral Assay with Mosquito Cells

The C6/36 and Aag2-AF5 cells were seeded at a density of 10^5^ cells per well in a 96-well tissue culture plate (BD Falcon). The cells were allowed to adhere overnight at 28 °C without CO_2_. Next, the dilution series of the compounds were prepared in the medium, after which the cultures were immediately infected with CHIKV at a multiplicity of infection (MOI) of 0.001. On day 3 post-infection, the supernatant was harvested, and intracellular RNA was extracted using the Cells-to-cDNA™ cell lysis buffer (Invitrogen, Thermo Fisher Scientific, Waltham, MA, USA).

The levels of infectious virus progeny in the supernatant were determined by end-point titrations on Vero cells. To this end, Vero cells were seeded at a density of 2.5 × 10^4^ cells per well in a 96-well tissue culture plate. The cells were allowed to adhere overnight. Next, 10-fold serial dilutions of the supernatant were prepared in the medium. At day 3 post-infection, the virus-induced cytopathic effect (CPE) was scored microscopically. The tissue culture infectious dose_50_/_mL_ (TCID_50_/_mL_), defined as the virus dose that would infect 50% of the cell cultures, was calculated using the Reed and Muench method [25].

Quantification of intracellular CHIKV genome copies was done by the one-step quantitative reverse transcription PCR (qRT-PCR) using the Applied Biosystems 7500 Fast Real-Time PCR System (Thermo Fisher Scientific) and primer and probe sequences targeting the nsP1 gene: 5′-CCGACTCAACCATCCTGGAT-3′, 5′-GGCAGACGCAGTGGTACTTCCT-3′, 5′-FAM-TCCGACATCATCCTCCTTGCTGGC-TAMRA [3]. For absolute quantification, standard curves were generated each run using 10-fold serial dilutions of a pCR4-TOPO-CHIKV-nsP1 plasmid. The antiviral activity of the compounds was also demonstrated in Vero cells, seeded at a density of 2.5 × 10^4^ cells per well in a 96-well tissue culture plate. The cells were allowed to adhere overnight at 37 °C and 5% CO_2_. The antiviral assay was performed as described for the mosquito-derived cells. Compound cytotoxicity, expressed as percentage cell viability, was determined at 3 days post-treatment for each cell line using 3-(4,5-dimethylthiazol-2-yl)-5-(3-carboxymethoxyphenyl)-2-(4-sulfophenyl)-2H-tetrazolium (MTS). Statistical significance was assessed with the Kruskal-Wallis test using the GraphPad Prism software (statistically significant means *p* < 0.05).

### 2.4. HPLC Analysis of T-705 Metabolites

C6/36, Aag2-AF5, and Vero cells were seeded in 25-cm^2^ flasks in a cell culture medium supplemented with 2% FBS and allowed to adhere overnight. Next, they were exposed to T-705 (1 mM) or DMSO (negative control) for 24 h. After this incubation period, the cells were trypsinized and counted, after which methanolic cell extracts were prepared and submitted to the anion-exchange HPLC analysis, as previously described [26]. The UV-detection was performed at 370 nm and the retention times for T-705-RMP, -RDP, and -RTP were 7.8, 13.5, and 21.3 min, respectively. The T-705 metabolites were quantified from integrated peak areas (i.p.a.), using chemically synthesized T-705-RMP and the ribonucleotides of T-1105, the non-fluorinated analogue of T-705, for standardization (all kindly provided by J. Huchting, University of Hamburg, Germany). These i.p.a. values were normalized to the total number of cells used for extraction. The lower limit of quantification was approximately 90 pmol/10^6^ cells. However, metabolites could still be detected at approximately 50 pmol/10^6^ cells (lower limit of detection) [26].

### 2.5. Aedes aegypti Rearing

*Ae. aegypti* Paea (Papeete, Tahiti, collected in 1994) were obtained via the Infravec2 consortium. Eggs were hatched in dechlorinated tap water. Following hatching, groups of ±200 larvae were transferred into trays containing 2 L of dechlorinated tap water and fed every day with a yeast tablet (Gayelord Hauser, Saint-Genis-Laval, France) until the pupae stage. Pupae were placed in cages of ±200 individuals each and the emerged adults were maintained at 28 ± 1 °C with a light/dark cycle of 16/8 h at 80% relative humidity and supplied with cotton soaked in a 10% sucrose solution.

### 2.6. CHIKV Infection and Compound Treatment of Aedes Mosquitoes

Mosquitoes were starved 24 h prior to infection. Seven-day-old female mosquitoes were orally infected for 30 min using an artificial membrane feeding system (Hemotek, UK). The blood meal contained washed fresh rabbit erythrocytes, 2.81 × 10^6^ PFU/mL of CHIKV 899, ATP (5 mM), and either T-705 in DMSO (600 µM), HCQ in sterile water (200 µM) or DMSO (0.8%) alone. Fully engorged females were cold-anesthetized and sorted to be either frozen immediately in PBS at −80 °C for viral input estimation or maintained for 48 h under controlled conditions (28 ± 1 °C, relative humidity of 80%, light/dark cycle of 16/8 h, supplied with a 10% sucrose solution). At 48 h post-infection, females which had finished blood digestion were collected and stored at −80 °C until further processing.

The infectious viral load per mosquito was determined by end-point titrations of mosquito homogenates. In brief, whole mosquitoes were homogenized individually in 300 µL PBS using bead disruption (2.8 mm Precellys). The supernatant from the mosquito homogenates was filtered using 0.8 µm MINI column filters (Sartorius, Göttingen, Germany). Titrations of filtered supernatant were performed on confluent Vero cells in 96-well plates. Infectious virus titers were calculated by the Reed and Muench method using the Lindenbach calculator and were expressed as TCID_50_/mosquito [25]. The infection rate (IR) was calculated as the proportion of blood-fed mosquitoes that contained CHIKV in their body as determined by end-point titrations. Statistical significance was assessed with the Mann-Whitney test using the GraphPad Prism software (ns = *p* > 0.05).

## 3. Results

**Figure 1:**
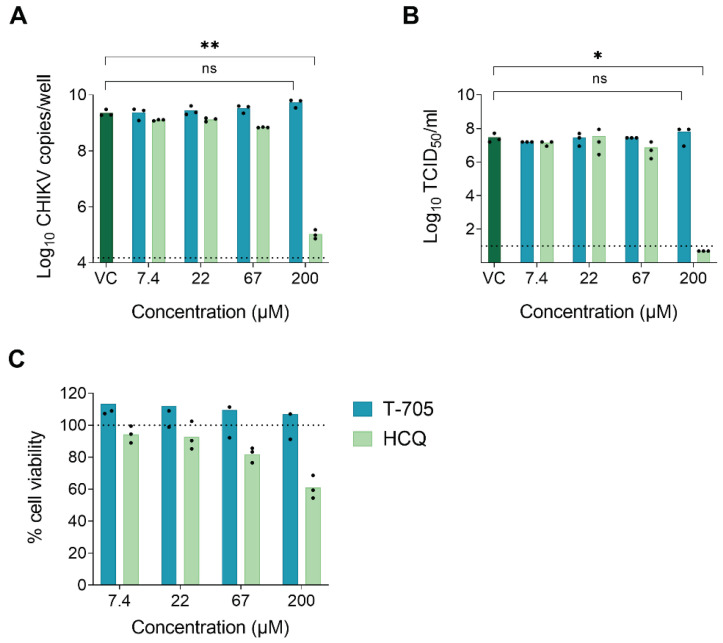
T-705 does not inhibit CHIKV in Aag2-AF5 cells. (**A**) Levels of intracellular viral RNA and (**B**) infectious virus progeny in the supernatant of Aag2-AF5 cells at day 3 post-infection following exposure to different compound concentrations. The dashed line represents the limit of detection. VC: Untreated virus control. (**C**) Compound-induced cytotoxic effects in Aag2-AF5 cells. The 100% cell viability indicates the absence of cytotoxic effects. Data shown are the results of at least two independent experiments; the bars show the mean value per condition. Statistically significant reductions in (**A**) CHIKV genome copies or (**B**) TCID_50_ after treatment with different compound concentrations as compared to the VC, were analyzed using the Kruskal-Wallis test (* *p* < 0.05; ** *p* < 0.005).

The metabolic activation pathway of T-705 to generate the active T-705-RTP form starts with ribophosphorylation by the HGPRT enzyme [6]. Since mosquitoes are considered as lacking a homologue of the HGPRT gene [20], we hypothesized that T-705 would be antivirally inactive in mosquito-derived cells and, consequently, unable to suppress virus replication in mosquitoes. To address this, we first determined the antiviral activity of T-705 against CHIKV in Aag2-AF5 cells (derived from *Ae. aegypti*). Since CHIKV does not induce a cytopathic effect (CPE) in mosquito cells, antiviral efficacy was assessed by the reduction in intracellular viral RNA, as quantified by qRT-PCR, and the reduction of infectious virus progeny in the supernatant, as determined by end-point titrations. A CHIKV inhibitor with an HGPRT-independent mechanism of action, hydroxychloroquine (HCQ), was used as a positive control. HCQ is considered to have a very similar mechanism of action as chloroquine (inhibition of endocytosis-mediated entry by increasing the endosomal pH [18]) and to be equipotent but less toxic [19,20]. T-705 was unable to inhibit CHIKV RNA replication in Aag2-AF5 cells at concentrations up to 200 µM. HCQ proved to be a modest inhibitor, resulting in a 4.3 log_10_ reduction in intracellular viral RNA at a concentration of 200 µM (Figure 1A). Similar results were obtained when quantifying the infectious virus progeny in the supernatant. At 200 µM, HCQ reduced the virus titer by 7.5 log_10_, whereas T-705 had no inhibitory effect (Figure 1B). T-705 did not cause cytotoxic effects at any of the concentrations tested in Aag2-AF5 cells. In contrast, HCQ resulted in a mean 40% reduction in cell viability at 200 µM (Figure 1C), suggesting that the observed antiviral effect might be (partially) due to cytotoxicity.

To confirm the lack of activity of T-705 in mosquito cells, antiviral assays were performed in another mosquito cell line, i.e., C6/36 cells (derived from *Ae. albopictus*). Again, T-705 was devoid of anti-CHIKV activity in this cell line (Figure S1A). Interestingly, HCQ did not have a cytotoxic effect in this cell line and was no longer able to inhibit CHIKV RNA replication (Figure S1A,B). In contrast to the results in mosquito cells, exposing Vero cells to 200 and 67 µM of T-705 resulted in 4.1 log_10_ and 3.1 log_10_ reductions in intracellular viral RNA, respectively (Figure S1C). HCQ proved to be a strong inhibitor of CHIKV RNA replication in Vero cells with a reduction of 4.2 log_10_ in intracellular viral RNA at a concentration of 22 µM (Figure S1C). T-705 showed no signs of cytotoxicity in Vero cells, whereas a concentration of 200 µM of HCQ resulted in a mean 37% reduction in cell viability (Figure S1D).

To investigate whether the lack of antiviral activity of T-705 in mosquito cells was due to inadequate metabolic activation, the metabolic profile of T-705 was determined in C6/36, Aag2-AF5, and Vero cells using anion-exchange HPLC [21]. Whereas, all three T-705 metabolites (i.e., T-705-RMP, -RDP, and -RTP) were detected in Vero cells that were exposed to 1 mM of T-705 for 24 h, none of these metabolites could be detected in the two mosquito cell lines (Table 1).

To corroborate the above findings in an in vivo model, *Ae. aegypti* Paea mosquitoes were infected by an artificial blood meal containing CHIKV in the presence of either T-705 (in DMSO), HCQ (in sterile water) or DMSO alone. The concentration of T-705 in the artificial blood meal was chosen based on data from a pharmacokinetic model in healthy human volunteers [27]. In this model, maintaining twice daily doses of T-705 of 1000, 1200, and 1800 mg resulted at a steady state in median plasma concentrations of 425, 530, and 856 µM. For HCQ, studies in healthy males using single doses of 200 mg in the form of oral tablets reported peak plasma levels of 0.12 µM after almost 4 h [28]. However, this concentration is well below the concentration of HCQ which is required to inhibit 50% of the virus (EC_50_) in Aag2-AF5 cells. Therefore, a higher concentration that showed an antiviral effect in Aag2-AF5 cells was selected.

**Figure 2:**
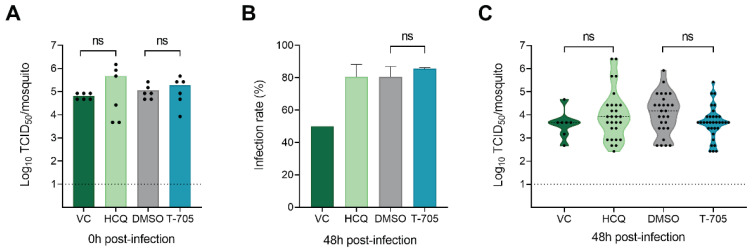
T-705 does not inhibit CHIKV in *Aedes aegypti* Paea mosquitoes. *Ae. aegypti* mosquitoes were fed with a blood meal containing CHIKV, CHIKV, and DMSO (0.8%), T-705 in DMSO (600 µM) or hydroxychloroquine in sterile water (HCQ, 200 µM). Engorged females were collected at 0 h post-infection and kept until 48 h post-infection. (**A**) Infectious virus titers in whole mosquitoes at 0 h post-infection, determined by end-point dilutions on Vero cells. (**B**) The infection rate represents the proportion of infected mosquitoes at 48 h post-infection among all engorged females tested (VC, *n* = 16; DMSO, *n* = 34; T-705, *n* = 41; HCQ, *n* = 34). (**C**) Infectious virus titers in whole mosquitoes at 48 h post-infection, determined by end-point dilutions on Vero cells. The data shown are the result of two independent experiments (for DMSO, T-705, HCQ). The VC data are the result of one experiment. The bars show the mean value (±SD) per condition. The dashed line (**A**,**C**) represents the limit of detection. The dotted lines in the violin plots (**C**) represent the median value per condition. Statistically significant differences between two groups were analyzed with the Mann-Whitney test (ns = *p* > 0.05).

The actual virus inoculum/mosquito, quantified by end-point titrations of whole engorged mosquitoes collected immediately after blood feeding, was similar in all four groups (Figure 2A). Two days after the blood feeding, the mosquito infection rates were approximately 80% for the DMSO and compound-treated groups and did not differ significantly between the DMSO and T-705-exposed groups (Figure 2B). The infection rate of the VC was lower, but this condition was only evaluated in a single experiment. The infectious virus titers in whole mosquitoes at 48 h post-infection were not significantly different between the DMSO and T-705 exposed groups or the VC and HCQ exposed groups (Figure 2C). In addition, no effect of the compounds on mosquito survival was observed at this time point. Together, these data indicate that neither of the compounds exerted an inhibitory effect on CHIKV replication in *Ae. aegypti* mosquitoes.

## 4. Discussion

This study is the first to evaluate the antiviral efficacy of T-705 in mosquito-derived cell lines, which are relevant for mosquito-borne viruses such as CHIKV. Our in vitro findings and metabolic activation data suggest that T-705 is not adequately activated in mosquito cells, which could explain why T-705 had no antiviral activity in the mosquitoes. However, it must be noted that the pharmacokinetic and -dynamic profile of T-705 in mosquitoes is not known. A previous study demonstrated that the average blood meal size of a mosquito was 3.2 µL [29]. The mean hemolymph volume of newly emerged adult females was estimated to be 336 nL/mosquito, decreasing by 43% after 2 weeks [30]. Assuming that all the molecules present in the blood meal would be absorbed into the hemocoel, concentrations of 600 µM T-705 would result in even higher concentrations in the hemolymph (>1 mM). However, it could be possible that the compound was excreted together with excess water and saline from the blood meal via the Malpighian tubes. Therefore, we did an attempt to assess the metabolic activation of T-705 in the mosquito body by HPLC. None of the T-705 metabolites could be detected in single or pooled mosquito body homogenates (data not shown). A further investigation into the pharmacokinetics and dynamics of T-705 in *Ae. aegypti* mosquitoes is thus needed to clarify the lack of antiviral activity.

To our knowledge, there are currently no reports on reference antiviral drugs that display robust antiviral activity against CHIKV in mosquito cells. A previous study reported on the ability of chloroquine (CQ) to inhibit CHIKV entry in C6/36 cells [31]. However, CQ did show signs of cytotoxicity which could lead to an overestimation of its antiviral potential. In this study, we evaluated the antiviral efficacy of its hydroxyl derivative, HCQ, as this compound is considered to be equipotent but less toxic. HCQ was unable to inhibit CHIKV in C6/36 cells but did show a modest antiviral activity against CHIKV in Aag2-AF5 cells. HCQ was not toxic in C6/36 cells, however, we did observe signs of cytotoxicity at a concentration of 200 µM in Aag2-AF5. These results suggest that the modest antiviral activity of HCQ in Aag2-AF5 cells might be (partially) due to compound-induced cytotoxicity. These data indicate that HCQ is not a suitable candidate to be used as a reference anti-CHIKV compound in mosquito cells. In contrast to the modest antiviral effect observed in vitro, HCQ was not active in *Ae. aegypti* mosquitoes. The lack of antiviral efficacy in vivo might be due to a pharmacokinetic and/or -dynamic failure in the mosquito. Another possibility is that viral entry in mosquito midgut cells in the context of a complex tissue in a live organism is different from viral entry in mosquito cell lines cultured in a monolayer. In the past, HCQ has shown the in vitro antiviral activity against several viruses including CHIKV, but efficacy in animal infection models was not confirmed [32]. Our results corroborate the discrepancies between the antiviral efficacy of this compound in cell culture and the corresponding in vivo infection model.

With the lack of vaccines and antiviral therapies for CHIKV, novel strategies are needed to supplement traditional vector-control methods that represent the main response. Such a new strategy could be the use of antiviral drugs to inhibit arboviral infection in the mosquito vector. Adult female mosquitoes become infected after taking a bloodmeal from a patient with sufficiently high viremia. Therefore, to inhibit arboviral infection of the mosquito vector with an antiviral drug, the drug should be taken up by the adult mosquito. There are two routes by which an adult mosquito could take up an antiviral drug. The first route is the tarsal route, i.e., uptake through the mosquito cuticle. A recent study demonstrated that tarsal exposure to antimalarial drugs resulted in potent *Plasmodium* blocking effects in *Anopheles* mosquitoes [33]. The second route to take up an antiviral drug is via oral ingestion, for example, through the ingestion of blood from a patient that is being treated with the drug or via attractive toxic sugar baits (ATSBs). When a patient receives the treatment with an antiviral drug, the drug will be present in the blood for a certain period of time. This renders the possibility that the mosquito midgut becomes exposed to the drug following a blood meal. For example, it was shown that the feeding of *Anopheles* mosquitoes on humans treated with the drug ivermectin resulted in significant mosquito lethality [34]. This indicates that a small molecule drug can be taken up via a blood meal and can exert a biological effect inside the mosquito. Ivermectin has been used in a phase 2 clinical trial as a therapeutic for dengue fever (ClinicalTrials.gov identifier: NCT03432442). The fact that this drug has both mosquitocidal and antiviral activity could be beneficial not only for vector control but also for limiting virus transmission. With regards to ATSBs, a recent review highlighted the potential negative impact of the insecticide in the baits on non-targeted (beneficial) insects such as honeybees [35]. If a small molecule antiviral drug could replace the insecticide, the bait could be considered more environmentally friendly with reduced accidental killing of beneficial insects.

Knowing the effect of antiviral drugs on virus replication in the mosquito vector could prove important to assess the true impact of antiviral therapies for arboviruses. The cross-species antiviral activity could be favorable since inhibition of the virus in the mosquito vector might prevent further transmission to vertebrate hosts. The evaluation of other drugs with a more potent anti-CHIKV activity is needed to determine whether exposure of mosquitoes to these compounds could effectively contribute to the prevention of arbovirus transmission.

## Figures and Tables

**Table 1:** Lack of T-705 metabolic activation in mosquito-derived cell lines.

Cell Line	pmol of Metabolite/10^6^ Cells		
	T-705-RMP	T-705-RDP	T-705-RTP
**Vero**	222 ± 131	170 ± 108	309 ± 122
**C6/36**	nd	nd	nd
**Aag2-AF5**	nd	nd	nd

Cells were treated with T-705 (1 mM) for 24 h, then submitted to cell counting, cell extraction, and HPLC analysis. Intracellular metabolites were quantified based on integrated peak areas (normalized for the number of cells), using chemically synthesized T-705-RMP and T-1105-ribonucleotides for standardization [21]. RMP, RDP, RTP: Ribonucleoside 5′-mono, -di, and -triphosphate; nd: Not detected.

## Supplementary Materials

The following are available online at https://www.mdpi.com/article/10.3390/microorganisms9050944/s1. Figure S1: T-705 inhibits CHIKV in mammalian Vero cells but not in *Ae. albopictus*-derived C6/36 cells.
