# Supplementary material for: Favipiravir Does Not Inhibit Chikungunya Virus Replication in Mosquito Cells and Aedes aegypti Mosquitoes
Source: Microorganisms. 2021 Apr 27;9(5):944. doi: 10.3390/microorganisms9050944 (PMC8145424; doi:10.3390/microorganisms9050944)
Supplement: Supplementary file 1 [file microorganisms-09-00944-s001.zip › microorganisms-1146495-supplementary.pdf]

microorganisms

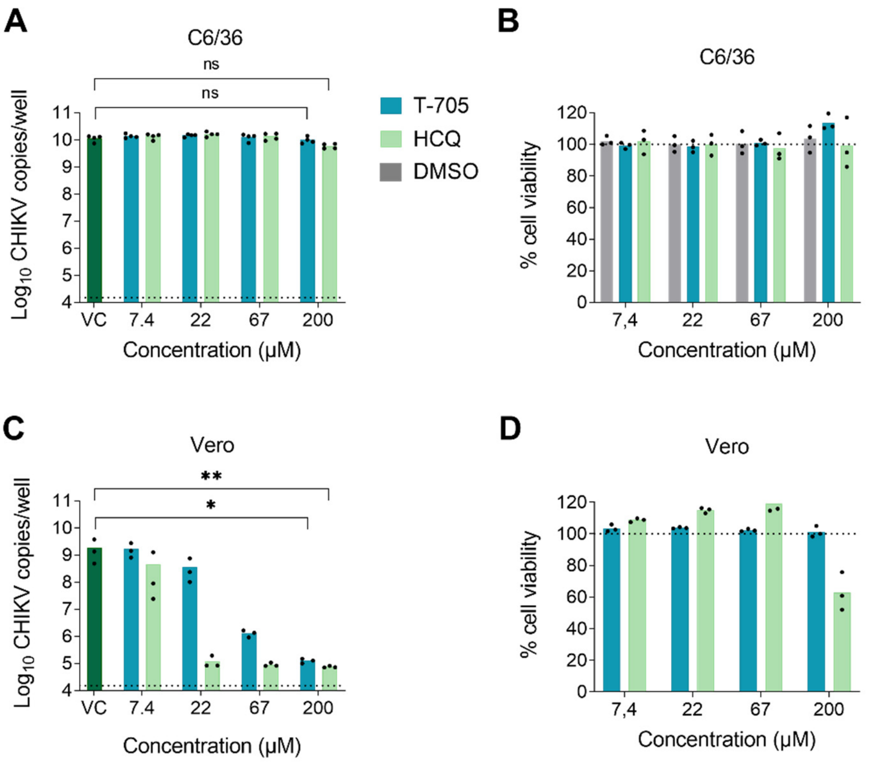

Figure S1. T-705 inhibits CHIKV in mammalian Vero cells, but not in *Ae. albopictus*-derived C6/36 cells. Levels of intracellular viral RNA in (**a**) C6/36 cells and (**c**) Vero cells at day 3 post-infection following exposure to different compound concentrations. Compound-induced cytotoxic effects in (**b**) C6/36 cells and (**d**) Vero cells. 100% cell viability indicates absence of cytotoxic effects. The DMSO treated cells in panel B were exposed to the same DMSO concentrations as the T-705 treated cells. Data shown are the results of at least three independent experiments; bars show the mean value per condition. VC, untreated virus control. (**a**,**c**) The dashed line in represents the limit of detection. Statistically significant reductions in (**a**) CHIKV genome copies or (**b**) $TCID_{50}$ after treatment with different compound concentrations as compared to the VC, were analyzed using the Kruskal-Wallis test (* $p < 0.05$; ** $p < 0.008$).
